# Supplementary material for: Digital technology and patient and public involvement (PPI) in routine care and clinical research—A pilot study
Source: PLoS One. 2023 Feb 3;18(2):e0278260. doi: 10.1371/journal.pone.0278260 (PMC9897511; doi:10.1371/journal.pone.0278260)
Supplement: S1 File — (DOCX) [file pone.0278260.s001.docx]

**SUPPLEMENTARY MATERIAL**

**APPENDIX 1 – Outline of study delivery**

The workshop opened with introductions of the research team and patient participants. The voluntary nature of responses was highlighted. A description of the proposed research study was provided, and participants were offered the opportunity to ask questions at any point.

There were three distinct phases to this study:

Planning _(3-months)

Steering group formed of authorship group

Sources of patient recruitment (NIHR, BHF, clinic)

Application to a small PPI fund (UCLH BRC)

Venue chosen - social distancing, AV facilities

Registration of interest form

Selection of participants from the those who responded - asked to confirm attendance – Use of a reserve list

Signposting and communication before the day

Day

AV check and room preparation

Face to face participants checked in. Mask wearing, social distancing, hand sanitiser.

Remote participants dialled in via Teams

Introductions of research team and patient participants

Description of proposed research study

Q&A

Verbal responses noted by scribe

Written responses to proforma

Close and thanks

Timeline for day

Evaluation

Participants invited to submit additional thoughts/comments/views

Formal post-PPI day feedback form distributed

Reimbursed in accordance with NIHR Involve guidance

Staff experience/ training

YC has completed previous PPI work relevant to research and presented his experiences at Heart Rhythm Congress 2021. YC and AH planned the session in advance with rehearsal of presentation timings and ability to scribe accurately. There was 30minutes setup and debrief

added to the timings. All content and material were reviewed by the authorship group.

**APPENDIX 2 – Slides used for PPI event**

**** ****

**** **
**

**** ****

**** ****

**Appendix 3 GRIPP2 Long form**

| **Table 1 \| GRIPP2 long form** |  |  |
| --- | --- | --- |
| **Section and topic** | **Item** | **Page** |
| **Section 1: Abstract of paper** |  |  |
| **1a: Aim** | **Report the aim of the study** | **2** |
| **1b: Methods** | **Describe the methods used by which patients and the public were involved** | **2** |
| **1c: Results** | **Report the impacts and outcomes of PPI in the study** | **2** |
| **1d:Conclusions** | **Summarise the main conclusions of the study** | **2** |
| **1e: Keywords** | **Include PPI, “patient and public involvement,” or alternative terms as keywords** | **1** |
| **Section 2: Background to paper** |  |  |
| **2a: Definition** | **Report the definition of PPI used in the study and how it links to comparable studies** | **4** |
| **2b: Theoretical underpinnings** | **Report the theoretical rationale and any theoretical influences relating to PPI in the study** | **4** |
| **2c: Concepts and theory development** | **Report any conceptual models or influences used in the study** | **n/a** |
| **Section 3: Aims of paper** |  |  |
| **3: Aim** | **Report the aim of the study** | **4** |
| **Section 4: Methods of paper** |  |  |
| **4a: Design** | **Provide a clear description of methods by which patients and the public were involved** | **4,5** |
| **4b: People involved** | **Provide a description of patients, carers, and the public involved with the PPI activity in the study** | **5** |
| **4c: Stages of involvement** | **Report on how PPI is used at different stages of the study** | **5** |
| **4d: Level or nature of involvement** | **Report the level or nature of PPI used at various stages of the study** | **5** |
| **Section 5: Capture or measurement of PPI impact** | |  |
| **5a: Qualitative evidence of impact             If applicable, report the methods used to qualitatively explore the impact of PPI in the study** | | **5-7** |
| **5b: Quantitative evidence of impact** | **If applicable, report the methods used to quantitatively measure or assess the impact of PPI** | **6-9** |
| **5c: Robustness of measure** | **If applicable, report the rigour of the method used to capture or measure the impact of PPI** | **n/a** |
| **Section 6: Economic assessment** |  |  |
| **6: Economic assessment** | **If applicable, report the method used for an economic assessment of PPI** | **n/a** |
| **Section 7: Study results** |  |  |
| **7a: Outcomes of PPI** | **Report the results of PPI in the study, including both positive and negative outcomes** | **5-9** |
| **7b: Impacts of PPI** | **Report the positive and negative impacts that PPI has had on the research, the individuals involved  (including patients and researchers), and wider impacts** | **5-9** |
| **7c: Context of PPI** | **Report the influence of any contextual factors that enabled or hindered the process or impact of PPI** | **8,9** |
| **7d: Process of PPI** | **Report the influence of any process factors, that enabled or hindered the impact of PPI** | **5-9** |
| **7ei: Theory development** | **Report any conceptual or theoretical development in PPI that have emerged** | **n/a** |
| **7eii: Theory development** | **Report testing of theoretical models, if any** | **n/a** |
| **7f: Measurement** | **If applicable, report all aspects of instrument development and testing (eg, validity, reliability, feasibility, acceptability, responsiveness, interpretability, appropriateness, precision)** | **n/a** |
| **7g: Economic assessment** | **Report any information on the costs or benefit of PPI** | **n/a** |
| **Section 8: Discussion and conclusions** |  |  |
| **8a: Outcomes** | **Comment on how PPI influenced the study overall. Describe positive and negative effects** | **9,10** |
| **8b: Impacts** | **Comment on the different impacts of PPI identified in this study and how they contribute to new knowledge** | **10** |
| **8c: Definition** | **Comment on the definition of PPI used (reported in the Background section) and whether or not you would suggest any changes** | **9** |
| **8d: Theoretical underpinnings** | **Comment on any way your study adds to the theoretical development of PPI** | **n/a** |
| **8e: Context** | **Comment on how context factors influenced PPI in the study** | **9,10** |
| **8f: Process** | **Comment on how process factors influenced PPI in the study** | **9,10** |
| **8g: Measurement and capture of PPI impact**  **8h: Economic assessment** | **If applicable, comment on how well PPI impact was evaluated or measured in the study** | **9,10** |
|  | **If applicable, discuss any aspects of the economic cost or benefit of PPI, particularly any suggestions for future economic modelling.** | **n/a** |
| **8i: Reflections/critical perspective** | **Comment critically on the study, reflecting on the things that went well and those that did not, so that others can learn from this study** | **10,11** |
