# Supplementary material for: Digital technology and patient and public involvement (PPI) in routine care and clinical research—A pilot study
Source: PLoS One. 2023 Feb 3;18(2):e0278260. doi: 10.1371/journal.pone.0278260 (PMC9897511; doi:10.1371/journal.pone.0278260)
Supplement: Attachment — Submitted filename: Response to reviewers.docx [file pone.0278260.s002.docx]

**Response to reviewers**

**Reviewer #1: This study contributes to the literature about hybrid PPI events to understand patients' points of view. However, it would make a more substantial contribution if a more comprehensive analysis of the qualitative findings were presented, not just presenting informants' quotations without further explanation of what they imply.**

**Thank you. We have added the following pieces of analysis:**

“These quotes were indicative of the enthusiasm for remote monitoring that the group had and their inquisitiveness about how such their BP data would be shared with their usual care team. However, there was an emphasis additionally placed on safeguarding required to mitigate against over-measurement and the possible anxiety that elevated readings could create, though this was balanced with points made about elevated readings being positively viewed, in terms of being a motivating factor to engage in lifestyle behaviours such as exercise to reduce BP.”

“This quote summarised the consensus view of the group in terms of how to balance the feasibility and safety of doing research studies of electronic alerts aimed at clinicians which seek to change their behaviour and downstream management strategies that can affect patients, In this regard, the group understood that thresholds for tolerating waived consent may vary case by case. There was a majority consensus for waived consent in the context of comparing existing non-pharmaceutical interventions such as fluid restriction versus no restriction, given the patient is the final arbiter of oral fluid intake.”

**Reviewer #2: The manuscript by Yang Chen and Colleagues is a well written and sound exploratory report, and may offer some relevant messages for assessing PPI. Patient and public involvement and engagement is gradually becoming part of standard cardiovascular and cancer research and an increasing number of statements are being released regularly to support it.**

**Thank you.**

**However, methods description and results presentation still have room for improvement and may be addressed. Please find my suggestions below.
- Little information is currently present regarding the steps that led to the final study cohort selection. It would be useful to know how the final study cohort was selected and derived (especially in light of the pre-event survey that Researchers conducted before the pilot study).**

**We have added the following:**

“A shortlist of 10 participants was purposively selected to generate a balanced group. This was based on protected characteristics such as age and gender, as well as pre-event confidence in using digital tools.”

**-Do Authors have more information regarding social variables of participants or do they have access to healthcare data?**

**We have added the following:**

“Other background information including social determinants of health were not collected in order to prioritise accessibility and ease of completion.”

**-From a practical point of view, it would be useful to have more information regarding study timeline, staff training, and study and interview duration.**

**We have added these relevant details to the supplementary material.**

**Response to editorial comments**

1. Please ensure that your manuscript meets PLOS ONE's style requirements, including those for file naming. The PLOS ONE style templates can be found at <https://journals.plos.org/plosone/s/file?id=wjVg/PLOSOne_formatting_sample_main_body.pdf> and 
<https://journals.plos.org/plosone/s/file?id=ba62/PLOSOne_formatting_sample_title_authors_affiliations.pdf>

2. Please amend your current ethics statement to address the following concerns:
a) Did participants provide their written or verbal informed consent to participate in this study?
b) If consent was verbal, please explain i) why written consent was not obtained, ii) how you documented participant consent, and iii) whether the ethics committees/IRB approved this consent procedure."

3. Please include your full ethics statement in the ‘Methods’ section of your manuscript file. In your statement, please include the full name of the IRB or ethics committee who approved or waived your study, as well as whether or not you obtained informed written or verbal consent. If consent was waived for your study, please include this information in your statement as well.

**We have added the following:**

“The pre-attendance short survey allowed prospective participants to provide written consent before the event, and verbal consent was taken on the day for use of non-attributable quotations that arose from discussions.”

“In accordance with the UK Health Research Authority decision tool on ethical requirements for research [11] and specific guidance from the National Institute for Health and Care Research on the ethics of PPI events, no approval from an ethics committee was sought.[12]”

4. Thank you for stating the following in the Acknowledgments Section of your manuscript:

"ADS is supported by a postdoctoral fellowship from THIS Institute, grants from NIHR and the UCL British Heart Foundation Research Accelerator. FWA and YC are supported by UCL Hospitals NIHR Biomedical Research Centre. AH is supported by a Health Education Eng-land Topol Digital Fellowship. The PPI event was kindly supported by funds from the NIHR UCLH BRC PPI bursary fund. The British Heart Foundation Heart Voices network were in-strumental in helping to recruit patients and we are grateful for the assistance of Chloe Gold-man in this regard. We are additionally grateful to Tom Lumbers for help with reviewing slides for the PPI day and contributing to the content and themes for the research element of the day. Lastly, we are grateful to all patients who expressed interest in the day and who kindly volunteered their time and views in helping to support this study and to inform the design of the two projects that have been discussed during this paper."

We note that you have provided funding information that is not currently declared in your Funding Statement. However, funding information should not appear in the Acknowledgments section or other areas of your manuscript. We will only publish funding information present in the Funding Statement section of the online submission form. 

Please remove any funding-related text from the manuscript and let us know how you would like to update your Funding Statement. Currently, your Funding Statement reads as follows:

"The author(s) received no specific funding for this work"

**We have removed the relevant sentences related to funding in the acknowledgements section.**

**In the funding statement, we have updated this to:**

This work was directly supported by a BRC PPI Bursary Award - BRC849/PPI/AH/104990
